# Quercetin enhances vitamin D_2_ stability and mitigate the degradation influenced by elevated temperature and pH value

**DOI:** 10.3906/kim-2103-5

**Published:** 2021-08-27

**Authors:** Sukkum Ngullie CHANG, Jeong Jun LEE, Hyun Jin KIM, Sun Chul KANG

**Affiliations:** 1 Department of Biotechnology, Daegu University, Gyeongsan Korea; 2 Naturetech Co. Ltd, Chungcheongnam-do Korea

**Keywords:** Vitamin D_2_, quercetin, stability, degradation, pH, temperature

## Abstract

Vitamin D_2_ (vit. D_2_) is a nutraceutical essentially needed for good health. However, it is susceptible to oxygen and high temperature. The use of natural products such as bioflavonoids possessing anti-degradative effect of vit. D_2_ degradation has not been described before. A combinational effect of vit. D_2_ with quercetin showed a positive effect and inhibited vit. D_2_ degradation when exposed to high temperature (50 ℃ and 75 ℃) at different time points. The results obtained revealed vit. D_2_ degradation was drastically increased with longer incubation under thermal treatment. However, quercetin and vit. D_2_ groups were able to significantly inhibit the degradation of vit. D_2_ and stabilize it, evaluated through the retention percentage. We also exposed vit. D_2_ at solutions with different pH values (1, 4, 5, 7, 10). Quercetin exerted vit. D_2_ anti-degradation at different pH values as well as under thermal pressure at different time points. Conclusively, quercetin can be an effective way to reduce temperature and pH induced degradation of vit. D_2_.

## 1. Introduction

Vitamins are very essential for the well-being and good health of humans and animals. They are not synthesized in the body of vertebrates and must be supplied to the body through our diets. It has been estimated that there will be about one billion people who would suffer from vitamin D deficiency in the near future [1]. Vitamins are classified as fat soluble and water soluble vitamins depending on their solubility to different solvents [2]. Vitamin D comes under the fat soluble vitamin and has two major forms, i.e. cholecalciferol (vitamin D_3_) can be synthesized in the body by 7-dehydrocholesterol (provitamin D_3_) upon sunlight exposure [3], whereas exposure to UV light converts ergosterol (provitamin D_2_) present in fungi to previtamin D_2_, which is isomerized to vit. D_2_ [4]. The structure of vit. D_2 _shows a double bond in the C22 position and also the presence of a methyl group in the C24 position, which vitamin D_3 _lacks. Vit. D_2 _and vit. D_3 _are biologically inert compounds, and the main biological activity comes from its metabolites. Vitamin D is mostly converted in the liver to 25-hydroxyvitamin D, and it is further hydroxylated from the kidneys to convert into a metabolically active form of vitamin, i.e. 1 alpha, 25 hydroxyvitamin D [5]. Since vitamin D are lipophilic, they possess very important biological properties. They can be stored in the body, especially in the liver. When it is required by particular part of the body, liver release some of these fat soluble vitamins into the bloodstream to deliver it on the cells or tissues in need. There has been increasing evidence in the recent years that deficiency of vitamin D contributes to multiple sclerosis and plays a crucial role in aggravating the disease progression [6], while excessive concentration of vitamin D in the body leads to numerous diseases namely hypercalcaemia, hypercalciuria and different kind of problems in the kidney [7–8]. Vitamin D has been known to support the absorption of calcium and is of vital importance in the development and formation of bones, in hypertension, anti-inflammation, immune modulation, cardiovascular disease, fertility, pregnancy, dementia and even anti-cancer activity [9–10].

The production of vitamin D on mass scale can be from sources such as, fish oil and lanolin. However, there are very few sources to find vit. D_2_, some of which are lichens [11], alfalfa shoots and primarily in some mushroom species belonging to genera Agaricus, Lentinula and Pleurotius, which has been reported to contain more amounts of vit. D_2_ after treatment with UV-radiation [12]. Mushroom contains ergosterol, low levels of vitamin D and sometimes vit. D_2_. By using gamma-irradiation and electron beams, ergosterol present in the mushroom undergoes a series of arrangement in its rings and form components such as previtamin D and an active form of vitamin D_2 _[13]. This is the most commonly used and low cost method, safe, and commercially feasible for production of vit. D_2_.

Quercetin is a very important bioflavonoid extracted from numerous plant source indicated in Supplemental Table 1. Quercetin (3,3’,4’,5,7-pentahydroxyflavone) comes from Latin word “Quercetum” meaning oak forest, and it belongs to class of flavanols possessing potent antioxidant properties [14–15]. Quercetin is yellow in color and is poorly soluble in hot water, insoluble in cold water and soluble in alcohol and lipids. Studies have demonstrated that flavonoids are effective against ant-inflammatory, anti-fungal, anti-viral and anti-cancer activities because of their free radical scavenging properties [16]. The radical reactions occur because of the oxidizing environment that we live in. Quercetin exerts its antioxidant potential through the effect on glutathione (GSH), signal transduction in pathways, enzymatic activity and reactive oxygen species (ROS) caused by different environmental and other factors. Quercetin maintains oxidative balance. In the structure of quercetin, we see five –OH groups (graphical abstract) on the sides of the phenyl rings, which are bound to the residues of amino acids on the active site of two enzymes. Hence, quercetin shows strong inhibition against enzymes involved in oxidative properties [17].

**Table 1:** Percentage of vit. D2 retention after thermal treatment at 75 ℃ for 2 h in the dark. Number of repetition for each treatment: n = 3. The data are represented here as the mean ± S.D, where *p < 0.05, **p < 0.01, ***p < 0.001, vit. D2 (5 μg) vs vit. D2 (5 μg) + quercetin (1 μg), vit. D2 (5 μg) vs vit. D2 (5 μg) + quercetin (2 μg), vit. D2 (5 μg) vs vit. D2 (5 μg) + quercetin (5 μg), vit. D2 (5 μg) vs vit. D2 (5 μg) + quercetin (10 μg). Statistical significance analysis was carried out through one-way analysis of variance (ANOVA) prism.

Vitamin and quercetin	75 °C, 2 h(% of vit. DD_2_ retention)
Vit D_2_ (5 μg)	49.56 ± 6.38
Vit D_2_ (5 μg) + Quercetin (1 μg)	72.77 ± 5.22
Vit D_2_ (5 μg) + Quercetin (2 μg)	79.23 ± 5.81
Vit D_2_ (5 μg) + Quercetin (5 μg)	86.74 ± 7.36
Vit D_2_ (5 μg) + Quercetin (10μg)	87.32 ± 3.65

Vit. D_2 _is a very unstable compound, and it undergoes degradation rapidly. The degradation may be caused by different factors, but it arises mostly from chemical substances and conditions promoting oxidation. Many research articles have shown that the temperature coefficients and other experimental data clearly indicate that the degradation of vit. D_2_ occurs because of a chemical reaction. Vit. D_2_ can decompose into different inactive forms when exposed to air and light [18]. The different conversion of vitamin Dupon exposure to light are suprasterols, trans-vitamin D, isotachysterol etc. Vitamin D upon exposure to thermal energy undergoes a reaction and converts into pyrovitamin D, isopyrovitamin D, previtamin D etc. Previtamin D can undergo further reactional changes as well [19]. All forms of vitamin D has been found to be susceptible to degradation, rendering its biological activity dysfunctional. These kinds of change are more widely observed in dry dispersed environment than storage in different emulsions where they remain stable. Numerous different methods for stabilizing the different forms of vitamin D have been screened. Some of the known methods include coatings to prevent contact with air. Many different coating agents such as dried whey with hydrogenated fats and calcium impart more stability than whey alone. Packing the vitamins in inert gases also efficiently delayed the rate of degradation of vitamins. In our study, we evaluated the role of a potent antioxidant quercetin to reduce or inhibit the degradation of vit. D_2_.

## 2. Materials and methods

### 2.1. Chemicals

Vit. D_2 _(ergocalciferol), quercetin, trifluoroacetic acid, tris-base, sodium acetate, sodium chloride, and hydrochloric acid were purchased from Sigma-Aldrich (St. Louis, MI, USA). Chloroform, ethanol, and methanol were purchased from merckmillipore (Frankfurter Strasse 250 Darmstadt, Germany). Hydrogen peroxide was purchased from Junsei chemical Co., Ltd (Nihonbashi-honcho, Chuo-ku, Tokyo). All other chemicals used for the study but not mentioned here were of the highest analytical grade.

### 2.2. Temperature stability test

For measuring the stability, vit. D_2 _at a concentration of 5 μg was added to different tubes and mixed with different concentration of quercetin (1, 2.5, 5, 10 μg). The tubes were covered with aluminum foils to protect from the degradation of vitamin from light. Next, the tubes were kept in an incubator adjusted with different temperatures (50 and 75 ℃) and incubated at different time points (30 min, 1 h, 2h, 3h, 6h). The incubation was carried out in the dark. After incubation, the amount of vit. D_2_ was analyzed by using trifluoroacetic acid method (TFA). The concentration of vit. D_2_ was converted into percentage of retention of vit. D_2_.

### 2.3. pH stability test

The solution was prepared using different pH buffers (1, 4, 5, 7, 10) for pH = 1 (0.1N HCl), pH = 4 (buffer solution, Samchun Chemical # B0618), pH = 5 (Sodium acetate buffer), pH = 7 (buffer solution, Samchun Chemical # B0630), pH = 10 (glycine-sodium hydroxide buffer (0.08M). To the solutions with different pH values, 5 μg of vit. D_2 _and 5–10 μg of quercetin was added into each solution in 1.5 mL tubes covered properly with aluminum foil to protect from degradation arising from exposure to light. The tubes were further vortexed thoroughly using a corning LSE vortex mixer. Next, the tubes were kept in an incubator adjusted to the standardized temperature (75 ℃) and incubated for 2h. The incubation was carried out in the dark. Finally, we estimated the amount of vit. D_2_ retention.

### 2.4. Solvent stability test

For measuring the stability of vit. D_2_ on ethanol solvent, vit. D_2 _(5 μg) with or without quercetin (5 and 10 μg) was added to varying concentration of (1, 5, 10, 50%) EtOH solvent. The tubes were further vortexed thoroughly using a corning LSE Vortex Mixer. Next, the tubes were placed in an incubator adjusted to the standardized temperature (75℃) and incubated for 2h. The incubation was carried out in the dark. The amount of vit. D_2_ retention was performed using trifluoroacetic acid method of analysis.

### 2.5. Determination of vitamin D2 by trifluoroacetic acid method

For determining the concentration of vit. D_2_, we followed the protocol from a previously published paper with some minor modifications [20]. Following the experiment, the samples were added with vit. D_2_ at a dosage of (0–10 μg), dissolved in an organic solvent ethanol contained in a 15 mL glass centrifuge tubes wrapped with aluminum foil to protect from light. The solvent was completely evaporated out under the vacuum at 35 ℃. The residue was mixed with 500 μL of chloroform and were mixed thoroughly. After the residue has been completely solubilized, 2 mL of trifluoroacetic acid (TFA) was added to each of the tubes, mixed and transferred into a 1 mL glass cuvette within 50 s from mixing. The optical density was zeroed in the instrument using a solvent of chloroform and trifluoroacetic in the ratio of (1:4, i.e 500 μL: 2 mL) before proceeding with the sample measurement. After measuring the absorbance at 490 nm within 50 s, 2 drops of hydrogen peroxide solution was added to the cuvette and mixed thoroughly. The absorbance was remeasured at 490 nm within 2 min ± 10 s after adding hydrogen peroxide. Latter measurement was subtracted from the former measurement and the concentration of vit. D_2_ from the standard curve was calculated by comparing the different concentrations of vit. D_2_ measured similarly. The colorimetric assay for detection of vit. D_2_ depends on the formed yellow color, and the concentration is determined by the intensity of the formed color, which is developed in reaction with trifluoroacetic acid. Accordingly, chloroform was added in order to intensify the maximum development of the yellow color. The presence of the volume of chloroform did not affect the stability of vit. D_2_.

### 2.6. Statistical analysis

Vit. D_2 _and quercetin were expressed as a mean ± standard deviation (SD) of the experiments. The statistical significance and the differences in the experimental groups were calculated by using one-way analysis of variance (ANOVA) with Tukey’s test and student’s *t* -test comparing all pair of columns, where * represents p - values < 0.05, ** represents p - values < 0.01, and *** represents p - values < 0.001.

## 3. Results and discussion

### 3.1. Optimization of vitamin D_2 _analysisassay and temperature induced degradation study

Various methods have been employed and standardized for measurement of vit. D_2 _and its metabolites, such as HPLC [21], mass spectrometry [22-23] etc. However, there is very few methods for determination of vit. D_2 _by using colorimetric assay. In our study, we carried the analysis for vit. D_2 _using TFA method. This method is based on the determination of vit. D_2 _after it is isomerized to isotachysterol in reaction with the addition of TFA. After optimization and calibration, we estimated the extent with which quercetin can protect the degradation of vit. D_2_. The results obtained revealed that, vit. D_2 _(5 μg) undergoing thermal treatment at 75℃ for 2h (Table 1) had a retention percentage of 49.56 ± 6.38 indicating more than 50% degradation of vit. D_2_. However, when vit. D_2 _(5 μg)together with varying concentration of quercetin (1, 2, 5, 10 μg) was exposed to similar thermal pressure for 2h, we observed that quercetin had the ability to stabilize vit. D_2 _from undergoing degradation. The retention percentage of vit. D_2_ increased dose-dependently (72.77 ± 5.22, 72.77 ± 5.22, 86.74 ± 7.36, 87.32 ± 3.65%) with an increase in quercetin concentration (Table 1) indicating that quercetin acted as a powerful antioxidant in maintaining oxidative balance and blocked the chemical reaction arising from the thermal treatment and stabilizing vit. D_2 _from degrading.

### 3.2. Effect of quercetin on temperature induced vitamin D2 degradation

Temperature induced degradation of vit. D_2 _has been extensively studied [16]. We evaluated the role of quercetin in protecting the degradation of vit. D_2_. Vit. D_2 _(5 μg) in combination withquercetin (5 and 10 μg) was exposed to a temperature of 50 ℃ and incubated at different time points (30 min, 1h, 2h, 3h, 6h). Results obtained from the study showed degradation to be below 50%. The retention percentage of vit. D_2 _were vit. D_2_ alone (5 μg): 81.93 ± 4.15, vit. D_2 _(5 μg)+ quercetin (5 μg) was 89.8 ± 5.73 and vit. D_2 _(5 μg)+ quercetin (10 μg) was 93.33 ± 1.80 for 50 ℃, 30 min incubation in the dark (Table 2). We noticed that vit. D_2 _degradation was not rapid even at 50 ℃ for 6h. The retention percentage were vit. D_2 _(5 μg): 62.58 ± 2.37, vit. D_2 _(5 μg)+ quercetin (5 μg): 84.43 ± 6.18 and vit. D_2 _(5 μg)+ quercetin (10 μg): 87.25 ± 7.67 for 50 ℃, 6h incubation in the dark (Table 2). In order to find a temperature where we can notice a 50% degradation of vitamin D_2_, we increased the temperature range to 75 ℃ and carried out the similar method of different time points study (Table 3). The percentage of vit. D_2 _(5 μg) retention was 77.31 ± 1.96, vit. D_2 _(5 μg)+ quercetin (5 μg) was 82.05 ± 2.32 and vit. D_2 _(5 μg)+ quercetin (10 μg) was 88.02 ± 0.93, at 75 ℃, 30 min, in the dark. When we incubated for 6h at 75 ℃ in the dark, we observed that vit. D_2_ (5 μg) had a massive degradation approximately around 66.5%. We observed that 2h incubation at 75 ℃ caused an approximate vit. D_2 _degradation of 50%, having a retention percentage of 49.56 ± 2.18. We standardized our entire experiment at 75 ℃, 2h incubation in the dark. Results showed that quercetin significantly inhibited the degradation of vit. D_2_,observablyvit. D_2 _(5 μg) was 49.56 ± 2.18 had a lesser retention percentage, whereas we observed a dose-dependent inhibition of degradation in quercetin supplemented groups, vit. D_2_ (5 μg)+ quercetin (5 μg) having 82.46 ± 1.12 retention percentage and vit. D_2 _(5 μg)+ quercetin (10 μg) had 85.40 ± 5.28 retention percentage (Table 3). Our results positively showed the protective role of the antioxidant quercetin mitigating vit. D_2 _degradation.

**Table 2:** Percentage of vit. D2 retention after thermal treatment at 50 ℃ incubated at different times in the dark. Number of repetition for each treatment: n = 3. The data are represented here as the mean ± S.D, where *p < 0.05, **p < 0.01, ***p < 0.001, ns is non-significant, vit. D_2_ (5 μg) vs vit. D_2_ (5 μg) + quercetin (5 μg), vit. D_2_ (5 μg) vs vit. D_2_ (5 μg) + quercetin (10 μg). Statistical significance analysis was carried out through one-way analysis of variance (ANOVA) prism.

Vitamin andQuercetin	0 °C, 0 min(% of vit. D_2_ retention)	50 °C, 30 min(% of vit. D_2_ retention)	50 °C, 1 h(% of vit. D_2_ retention)	50 °C, 2 h(% of vit. D_2_ retention)	50 °C, 3 h(% of vit. D_2_ retention)	50 °C, 6 h(% of vit. D_2_ retention)
Vit D_2_ (5 μg)	100 ± 5	94.67 ± 4.15	92.97 ± 3.78	85.89 ± 4.72	78.82 ± 3	62.58 ± 2.37
Vit D_2_ (5 μg) +Quercetin(5 μg)	100 ± 7	96.13 ± 1.52	95.49 ± 3.05	90.27 ± 5.12	87.38 ± 1.73	84.43 ± 6.18
Vit D_2_ (5 μg) +Quercetin (10μg)	100 ± 5	97.33 ± 1	95.62 ± 2.78	91.43 ± 6	88.45 ± 4.58	87.25 ± 7.67

**Table 3:** Percentage of vit. D_2_ retention after thermal treatment at 75 ℃ incubated at different time points in the dark. Number of repetition for each treatment: n = 3. The data are represented as the mean ± S.D. from 3 independent experiments where *p < 0.05, **p < 0.01, ***p < 0.001, vit. D_2_ (5 μg) vs vit. D_2_ (5 μg) + quercetin (5 μg), vit. D_2_ (5 μg) vs vit. D_2_ (5 μg) + quercetin (10 μg). Statistical significance analysis was carried out through one-way analysis of variance (ANOVA) prism.

Vitamin and Quercetin	75 °C , 30 min(% of vit. D_2_ retention)	75 °C , 1 h(% of vit. D_2_ retention)	75 °C , 2 h(% of vit. D_2_ retention)	75 °C , 3 h(% of vit. D_2_ retention)	75 °C , 6 h(% of vit. D_2_ retention)
Vit D_2_ (5 μg)	77.31 ± 1.96	62.33 ± 3.93	49.56 ± 2.18	38.82 ± 2.49	33.82 ± 1.44
Vit D_2_ (5 μg) +Quercetin (5 μg)	82.05 ± 2.32	85.46 ± 4.72	82.46 ± 1.12	83.67 ± 1.49	79.67 ± 2.52
Vit D_2_ (5 μg) +Quercetin (10μg)	88.02 ± 0.93	86.60 ± 7.46	85.40 ± 5.28	86.51 ± 1.84	82.514 ± 4.36

### 3.3. Effect of quercetin on temperature and pH induced vitamin D2 degradation

We evaluated the influence of quercetin in protecting vit. D_2_ from degrading under constant thermal pressure and under different pH solutions. According to the previous study by other researchers, vit. D_2_ has been found to be stable at 20 ℃ in lemon juice maintained at a pH value of 2.5–4.5. The percentage of vit. D_2_ retention was around 99 ± 3% and 97 ± 3 % [24]. Consistent with the previous results, our results obtained showed that vit. D_2 _was mostly stable when incubated at different pH solution (pH = 4, 5, 7, 10) for 1h at RT (room temperature). However, at pH = 1, the results showed vit. D_2 _(5 μg) had a retention percentage of 83.63 ± 1.17, whereas vit. D_2_ (5 μg)+ quercetin (5 μg) had a retention of 89.71 ± 0.44 and vit. D_2 _(5 μg)+ quercetin (10 μg) of 90.22 ± 0.80 (Supplemental Table 2). No such significant difference in vit. D_2 _degradation was observed at other pH groups. Next, we evaluated the degradation of vit. D_2 _in different pH solutions at a temperature of 75 ℃ and 2h incubation in the dark. At pH = 1, under elevated temperature of 75 ℃ and 2h incubation in the dark, we observed maximum degradation in vit. D_2_ alone group in comparison to quercetin treatment groups. Vit. D_2_ (5 μg) had a retention percentage of 28.59 ± 0.48, whereas vit. D_2 _(5 μg)+ quercetin (5 μg) had a retention of 96.04 ± 5.19 and vit. D_2 _(5 μg)+ quercetin (5 μg) of 97.96 ± 3.46. The stability of vit. D_2 _was observably stable when incubated in combination with quercetin, which was consistently observed at all pH groups. The retention percentage of vit. D_2 _was comparatively better at pH = 4 and 5, 75 ℃ and 2h incubation (65.77 ± 2.96 and62.44 ± 2.88); however, it reduced with an increase of pH to 7 (46.46 ± 3.93) and 10 (45.42 ± 3.20). The percentage of vit. D_2 _retention for different concentration of quercetin was significantly higher in comparison to only vit. D_2 _group. At pH = 1, 4, 5, 7 and 10, the retention percentage of vit. D_2 _for vit. D_2 _(5 μg)+ quercetin (5 μg) group were 89.71 ± 0.44, 98.03 ± 0.19, 96.30 ± 2.83, 97.90 ± 0.22 and 98.03 ± 0.19, whereas for vit. D_2 _(5 μg)+ quercetin (10 μg) group, the retention percentages were 90.22 ± 0.80, 97.96 ± 0.22, 96.04 ± 0.55, 97.39 ± 0.48 and 95.92 ± 0.19 (Table 4). These results further elucidated the protective effect of quercetin against pH and temperature induced vit. D_2 _degradation.

**Table 4:** Percentage of vit. D_2_ retention at different pH and thermal treatment at 75 ℃ incubated at different time points in the dark. Number of repetition for each treatment: n = 3. The data are represented as the mean ± S.D. from 3 independent experiments where *p < 0.05, **p < 0.01, ***p < 0.001, vit. D_2_ (5 μg) vs vit. D_2_ (5 μg) + quercetin (5 μg), vit. D_2_ vs vit. D_2_ (5 μg) + quercetin (10 μg). Statistical significance analysis was carried out through one-way analysis of variance (ANOVA) prism.

Vitamin and Quercetin	pH = 1, 75 °C, 2 h(% of vit. D_2_ retention)	pH = 4, 75 °C , 2 h(% of vit. D_2_ retention)	pH = 5, 75 °C , 2 h(% of vit. D_2_ retention)	pH = 7, 75 °C , 2 h(% of vit. D_2_ retention)	pH = 10, 75 °C , 2 h(% of vit. D_2_ retention)
Vit D_2_ (5 μg)	28.59 ± 0.48	65.77 ± 2.96	62.44 ± 2.88	46.46 ± 6	45.42 ± 5
Vit D_2_ (5 μg) +Quercetin(5 μg)	96..04 ± 5.19	93.96 ± 4.16	91.96 ± 1.73	85.73 ± 5	92.45 ± 1.52
Vit D_2_ (5 μg) +Quercetin (10μg)	97.96 ± 3.46	95.90 ± 4	96.56 ± 3.05	91.2 ± 5	97.94 ± 2.88

### 3.4. Effect of solvent and temperature induced vitamin D2 degradation

We evaluated the solvent stability of vit. D_2 _in ethanol. We prepared vit. D_2_ at different percentage of ethanol (1, 5, 10 and 50%) and exposed it to a temperature of 75 ℃ and 2h incubation in the absence of light to avoid any photochemical reaction. After incubation, the samples were analyzed for the degradation of vit. D_2_. Our results indicated that vit. D_2 _was stable at 1%, 5%, 10% and 50% EtOH solution having a retention percentage of 84.12 ± 4.72, 83.72 ± 3.10, 83.36 ± 2.58 and 82.03 ± 3.51 in comparison to only vit. D_2 _group having a retention percentage of 45.78 ± 3.1 and vit. D_2 _(5 μg)+ quercetin (5 μg) group 83.17 ± 3.24 (Table 5). Our conclusion from this finding was as follows: Ethanol at concentration of below 50% did not have significant effect on the degradation and stability of vit. D_2_; however, ethanol concentration above 50% hindered the yellow color development, and no accurate measurement of vit. D_2 _quantification was obtained.

**Table 5:** Percentage of the retention of vit. D_2_ at 75℃, 2h and different concentration of ethanol. Number of repetition for each treatment: n = 3. The data are represented as the mean ± S.D. from 3 independent experiments where *p < 0.05, **p < 0.01, ***p < 0.001, ns is non-significant, vit. D_2_ (5 μg) vs other groups. Statistical significance analysis was carried out through one-way analysis of variance (ANOVA) prism and was non-significant for all groups.

Vitamin and Quercetin	75 °C , 2 h(% of vit. D2 retention)
Vit D_2_ (5 μg)	45.78 ± 3.17
Vit D_2_ (5 μg) +Quercetin(5 μg)	86.17 ± 3.24
Vit D_2_ (5 μg) +Quercetin(5 μg) EtOH 1%	84.12 ± 4.72
Vit D_2_ (5 μg) +Quercetin(5 μg) EtOH 5%	83.72 ± 3.10
Vit D_2_ (5 μg) +Quercetin(5 μg) EtOH 10%	83.36 ± 2.58
Vit D_2_ (5 μg) +Quercetin(5 μg) EtOH 50%	82.03 ± 3.50

## 4. Conclusion

In the present study, we investigated the role of bioflavanol quercetin in stabilizing vit. D_2_ degradation induced by high temperature and different pH buffers. Numerous publications have been studied thoroughly in the current study to find a suitable candidate and its role in inhibiting vit. D_2_ degradation. As we know, vit. D_2 _is very unstable and renders its biological activity, dysfunctional upon oxidation with air and light, and is prone to atrophy while using different vitamin D forms. Several methods have been described in the literature such as using of emulsions and oils to make them stable. However, using a natural product from a plant source having a potent free radical scavenging effect has not been described before. We observed combinational effect of vit. D_2_ with quercetin to be more stable and was able to significantly reduce vit. D_2_ degradation when exposed to high temperature (50 ℃ and 75 ℃) at different time points. Quercetin also exerted vit. D_2_ anti-degradation at different pH buffers ranging from 1 to 10, hence, substantiated the role of quercetin as an alternative source for vit. D_2 _stability. However, more studies need to be carried out with the use of different flavanoids such as quercetin for stability of different highly susceptible vitamin D forms such as vit. D_2_.

Supplementary Materials
