# Supplementary material for: Quercetin enhances vitamin D2 stability and mitigate the degradation influenced by elevated temperature and pH value
Source: Turk J Chem. 2021 Aug 27;45(4):1155–61. doi: 10.3906/kim-2103-5 (PMC8517607; doi:10.3906/kim-2103-5)
Supplement: Supplementary file 1 — Supplementary Materials [file turkjchem-45-1155-sup001.pdf]

**Supplemental Table 1.** Source of quercetin from different plants.

| Binomial nomenclature of plants possessing quercetin | Plant family | Therapeutic properties and Uses |
|---|---|---|
| *Allium cepa (red onions)* | *Liliaceae* | Potent antioxidant, regulator of immune system, cardioprotection, anti-cancer activity. |
| *Allium fistulosum* | *Amaryllidaeceae* | Popular food ingredient |
| *Apium graveolens* | *Apiaceae* | Anti-inflammation, anti-bacterial, reduce blood pressure and blood glucose |
| *Asparagus officinalis* | *Asparagaceae* | Anti-neoplastic, anti-ulcer |
| *Brassica oleracea var.* | *Brassicaceae* | An edible plant, reduce blood glucose level, cholesterol, neuropathy, reduces stroke |
| *Camellia sinensis* | *Theaceae* | Potent analgesic, anti-diabetic, anti-viral, bronchodilator |
| *Centella asiatica* | *Apiaceae* | Wound healing activity |
| *Capparis spinosa* | *Capparaceae* | Anti-atherosclerotic, disinfectants activity |
| *Hypericum hircinum* | *Hypericaceae* | Potent antioxidant activity |
| *Lactuca sativa* | *Asteraceae* | Anemia, iron deficiency treatment, osteoporosis |
| *Malus domestica* | *Rosaceae* | cardioprotection, anti-cancer activity |
| *Prunus Avium* | *Rosaceae* | Astringent, diuretic, potent tonic activity |
| *Solanum lycopersicum* | *Solanaceae* | Food supplement and salads |
| *Vaccinum oxycoccus* | *Ericaceae* | Treatment of gall bladder and urinary tract infections |

**Supplemental Table 2.** Percentage of the retention of vit. $D_2$ different pH in RT. Vit. $D_2$ (5μg) and different doses of quercetin (5μg and 10μg). Number of repetition for each treatment: n = 3. The data are represented as the mean ± S.D. from 3 independent experiments where *p < 0.05, **p < 0.01, ***p < 0.001, ns is non-significant, vit. $D_2$ (5 μg) vs other groups. Statistical significance analysis was carried out through one-way analysis of variance (ANOVA) prism and was non-significant for all groups.

| Vitamin and Quercetin | pH = 1, RT, 1 h (% of vit. $D_2$ retention) | pH = 4, RT, 1 h (% of vit. $D_2$ retention) | pH = 5, RT, 1 h (% of vit. $D_2$ retention) | pH = 7, RT, 1 h (% of vit. $D_2$ retention) | pH = 10, RT, 1 h (% of vit. $D_2$ retention) |
|---|---|---|---|---|---|
| Vit $D_2$ (5 μg) | 83.63 ± 1.17 | 88.62 ± 3.21 | 97.96 ± 0.22 | 97.96 ± 0.22 | 98.09 ± 0.48 |
| Vit $D_2$ (5 μg) + Quercetin(5 μg) | 89.71 ± 0.44 | 98.03 ± 0.19 | 96.30 ± 2.83 | 97.90 ± 0.22 | 98.03 ± 0.19 |
| Vit $D_2$ (5 μg) + Quercetin (10μg) | 90.22 ± 0.80 | 97.96 ± 0.22 | 96.04 ± 0.55 | 97.39 ± 0.48 | 95.92 ± 0.19 |
